# P-1946. Candida auris in Metropolitan Atlanta: Risk Factors for Mortality and Microbiologic Persistence

**DOI:** 10.1093/ofid/ofaf695.2114

**Published:** 2026-01-11

**Authors:** Lucy S Witt, Susan M Ray, Jessica Howard-Anderson, Stepy Thomas, Shanita Shack, Amy K Tunali, Scott Fridkin

**Affiliations:** Emory University, Atlanta, GA; Emory University School of Medicine, Atlanta, Georgia; Emory University, Atlanta, GA; Georgia Emerging Infections Program, Atlanta, Georgia; Georgia Emerging Infections Program, Atlanta, Georgia; Emory University - School of Medicine, Bentonville, Arkansas; emory university, Atlanta, Georgia

## Abstract

**Background:**

*Candida auris* is a highly transmissible yeast, and invasive infections are associated with high mortality. Using data from the Georgia Emerging Infections Program (GAEIP), we describe the characteristics of patients with *C. auris* fungemia in metropolitan Atlanta since the first area case was identified in 2022 and describe risk factors for in-hospital mortality and microbiologic persistence.

**Table 1.**
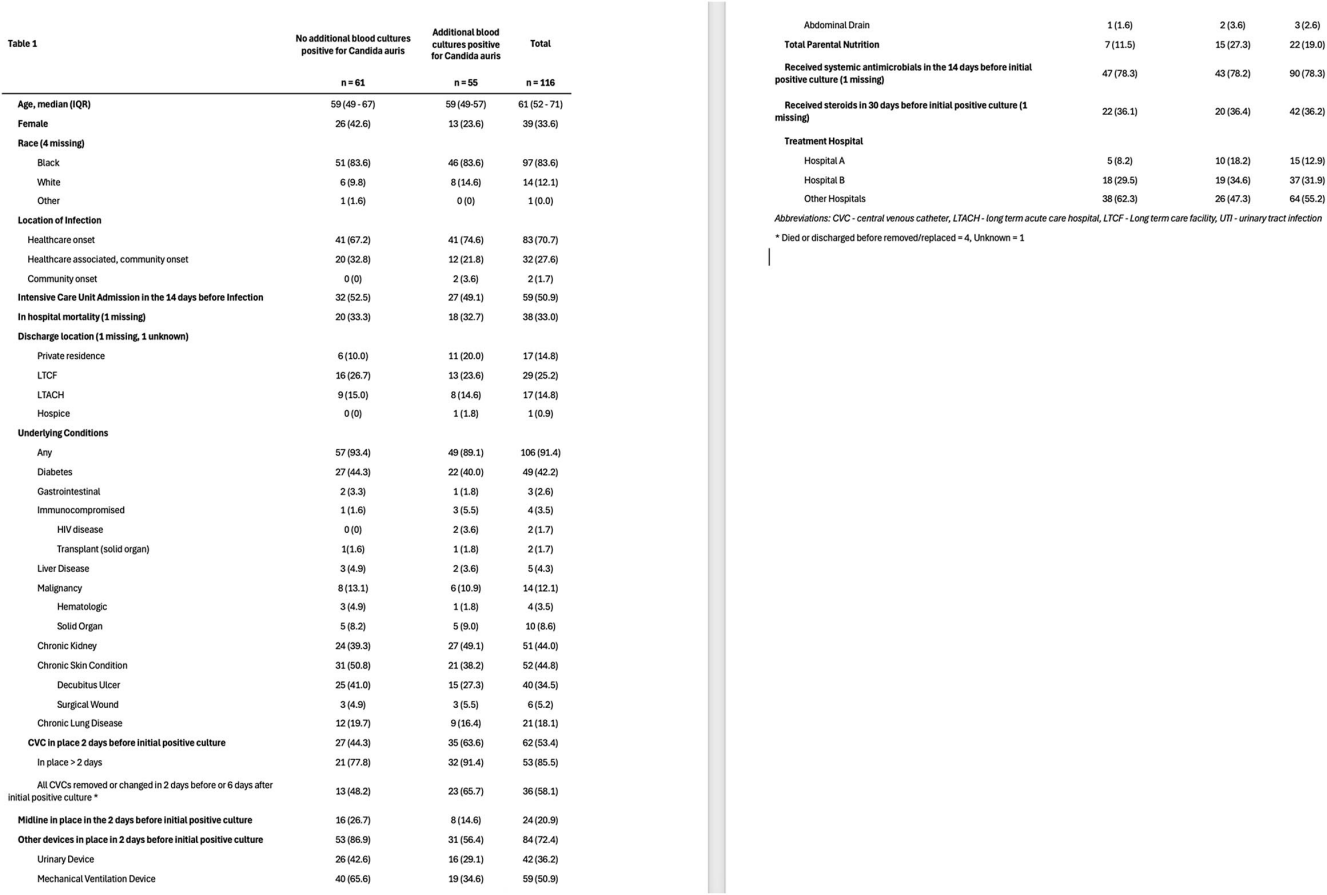
Basic demographics and clinical characteristics of patients with C. auris fungemia by microbiological persistence. All values are n (%) unless otherwise specified. Demographics and clinical characteristics according to in-hospital mortality. All values are n (%) unless otherwise specified.

**Methods:**

The EIP is an active laboratory- and population-based surveillance system through a collaboration with the Centers for Disease Control and Prevention. This study included all patients with *C. auris* fungemia in the GAEIP catchment area from 2022 - 2024. Only residents of the area were eligible for inclusion and only the incident infection was considered. Clinical characteristics were evaluated for association with microbiological persistence, defined as *C. auris* in the blood at any time in the 29-days after the incident infection, and for in-hospital mortality. Multivariable regression was performed to assess risk of mortality for patients with microbiological persistence while controlling for ICU admission, central venous catheter (CVC) use, chronic kidney disease, and treatment facility.

**Table 2.**
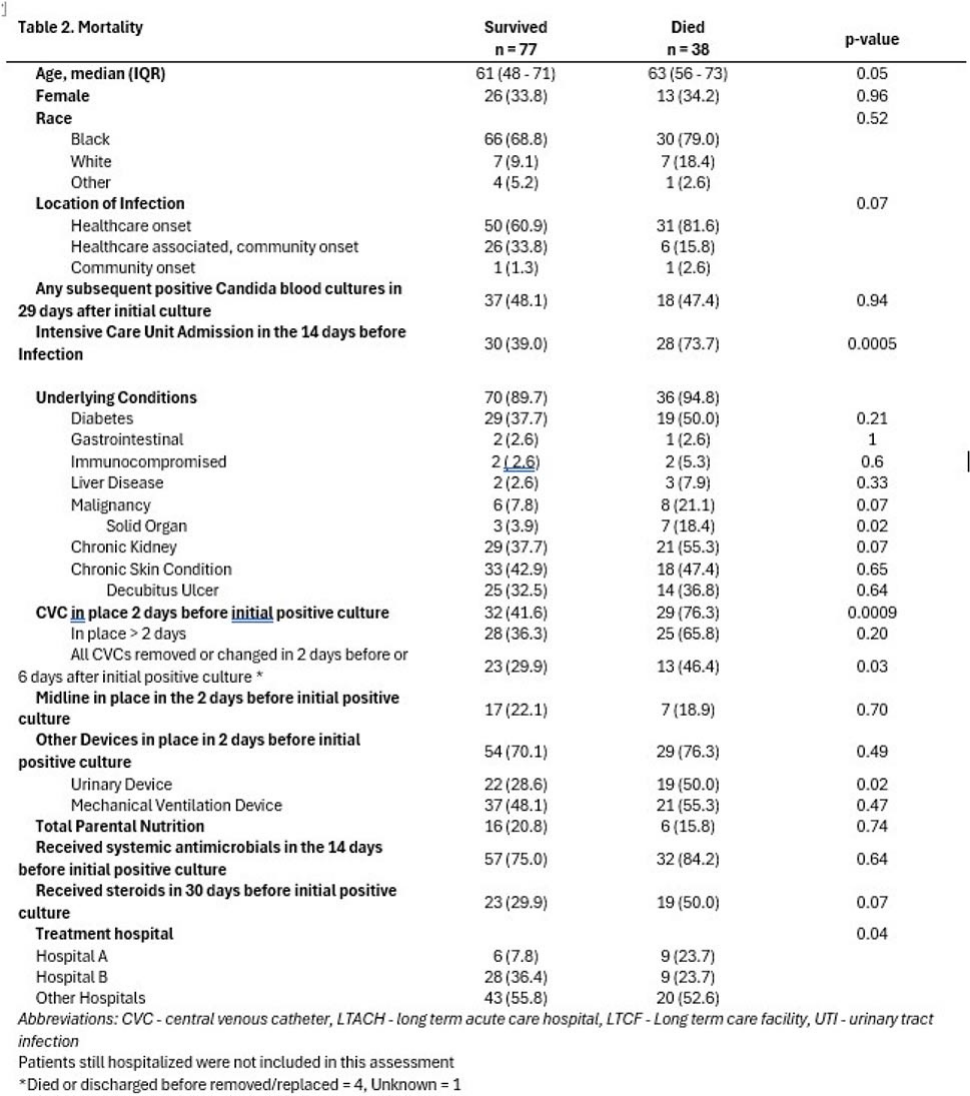
Multivariable regression model examining microbiological persistence and in-hospital mortality.

**Results:**

Between 2022 and 2024, 116 unique cases of *C. auris* fungemia were identified. Almost half of patients had microbiologic persistence and half had a CVC in place prior to infection, however this was more prevalent in patients with microbiologic persistence (63.6% v 44.3%, Table 1). In-hospital mortality was 32.8% and did not differ between patients with microbiological persistence and those without (Table 1). Older age, ICU, solid organ malignancy, urinary device or CVC in place, and treatment hospital were associated with in-hospital mortality (Table 2). On multivariable analysis microbiological persistence was not associated with in-hospital mortality, while ICU admission and CVC were (Table 3).

**Conclusion:**

In this analysis of *C. auris* fungemia in metro-Atlanta, we found that traditional risk factors were associated with mortality. Future studies will examine the relationship of specific antifungals with mortality and microbiologic persistence.

**Disclosures:**

Lucy S. Witt, MD, MPH, MSc, Merck & Co: Grant/Research Support

